# A Generic, Scalable, and Rapid Time-Resolved Förster Resonance Energy Transfer-Based Assay for Antigen Detection—SARS-CoV-2 as a Proof of Concept

**DOI:** 10.1128/mBio.00902-21

**Published:** 2021-05-18

**Authors:** Juuso Rusanen, Lauri Kareinen, Leonora Szirovicza, Hasan Uğurlu, Lev Levanov, Anu Jääskeläinen, Maarit Ahava, Satu Kurkela, Kalle Saksela, Klaus Hedman, Olli Vapalahti, Jussi Hepojoki

**Affiliations:** a University of Helsinki, Faculty of Medicine, Medicum, Department of Virology, Helsinki, Finland; b University of Helsinki, Faculty of Veterinary Medicine, Department of Veterinary Biosciences, Helsinki, Finland; c HUS Diagnostic Center, HUSLAB, Clinical Microbiology, University of Helsinki, Helsinki, Finland; d Helsinki University Hospital, Helsinki, Finland; e University of Zürich, Vetsuisse Faculty, Institute of Veterinary Pathology, Zürich, Switzerland; Medical School, National and Kapodistrian University of Athens

**Keywords:** antigen test, COVID-19, SARS-CoV-2, TR-FRET, mix and read, rapid diagnostic test

## Abstract

The ongoing coronavirus disease 2019 (COVID-19) pandemic has seen an unprecedented increase in the demand for rapid and reliable diagnostic tools, leaving many laboratories scrambling for resources. We present a fast and simple assay principle for antigen detection and demonstrate its functionality by detecting severe acute respiratory syndrome coronavirus 2 (SARS-CoV-2) antigens in nasopharyngeal swabs. The method is based on the detection of SARS-CoV-2 nucleoprotein (NP) and S protein (SP) via time-resolved Förster resonance energy transfer (TR-FRET) with donor- and acceptor-labeled polyclonal anti-NP and -SP antibodies. Using recombinant proteins and cell culture-grown SARS-CoV-2, the limits of detection were established as 25 pg of NP or 20 infectious units (IU) and 875 pg of SP or 625 IU. Testing reverse transcription-PCR (RT-PCR)-positive (*n* = 48, with cycle threshold [*C_T_*] values from 11 to 30) or -negative (*n* = 96) nasopharyngeal swabs demonstrated that the assay yielded positive results for all samples with *C_T_* values of <25 and for a single RT-PCR-negative sample. Virus isolation from the RT-PCR-positive nasopharyngeal swabs showed a strong association between the presence of infectious virus and a positive antigen test result. The NP-based assay showed 97.4% (37/38) sensitivity and 100% (10/10) specificity in comparison with virus isolation and 77.1% (37/48) sensitivity and 99.0% (95/96) specificity in comparison with SARS-CoV-2 RT-PCR. The assay is performed in a buffer that neutralizes SARS-CoV-2 infectivity, and the assay is relatively simple to set up as an “in-house” test. Here, SARS-CoV-2 served as the model pathogen, but the assay principle is applicable to other viral infections, and the test format could easily be adapted to high-throughput testing.

## INTRODUCTION

The ongoing coronavirus disease 2019 (COVID-19) pandemic has by December 2020 claimed almost 1.5 million lives globally, with over 60 million confirmed infections. To manage the disease, accurate diagnostic tools are of key importance. Detection of the causative agent, severe acute respiratory syndrome coronavirus 2 (SARS-CoV-2), or its parts is the cornerstone of diagnosis, as the disease presentation is often indistinguishable from those of other respiratory infections. The mainstay of COVID-19 diagnosis is reverse transcription-PCR (RT-PCR) testing, done typically from a nasopharyngeal swab (NPS), while oropharyngeal or midturbinate swabs as well as salivary samples are also in use. Alternatively, the less-labor-intensive antigen detection tests are also increasingly being deployed. Antigen tests tend to be specific but analytically less sensitive than RT-PCR. RT-PCR can detect viral nucleic acid even after the infectious virus has waned, with the individual at this time being unlikely to pose a transmission risk (1–3). Evidence suggests that antigen testing may correlate with the recovery of infectious virus better than a binary RT-PCR (4). Frequent antigen testing has been proposed as an alternative approach in reducing the community transmission of SARS-CoV-2 (5).

SARS-CoV-2 is an enveloped positive-sense single-stranded RNA [(+)ssRNA] virus of the genus *Betacoronavirus* subfamily *Orthocoronavirinae* in the family *Coronaviridae* of the order *Nidovirales*. It contains four structural proteins. The nucleoprotein (NP) forms a ribonucleoprotein complex with the 30-kb nonsegmented viral genome. The envelope (E) and membrane (M) proteins are embedded in the envelope, as is the spike protein (SP), protruding from the virion surface and generating large surface projections termed the corona. The SP undergoes processing to yield S1, which contains the receptor-binding domain (RBD) initially attaching the virus to angiotensin-converting enzyme 2 (ACE-2) on the host cell membrane, and S2, which mediates virus-cell fusion. In response to the pandemic, dozens of commercial SARS-CoV-2 antigen tests are available, predominantly of a lateral flow or enzyme immunoassay type. Most target NP as the analyte (6). Of the seven antigen tests having received emergency use authorization (EUA) from the U.S. Food and Drug Administration (FDA) by December 2020, six target NP, and one targets SP (7).

Over the past few years, we have actively employed time-resolved Förster resonance energy transfer (TR-FRET) as the basis of rapid homogeneous “mix-and-read” immunoassays for antibody detection (8–14). FRET occurs when a donor and an acceptor fluorophore are in proximity, whereby the excited donor transfers energy to the acceptor, which then emits a photon at a distinct wavelength. The closer the donor and acceptor are, the more frequent the energy transfer is, with a 50% efficiency typically being achieved at a distance of 15 to 60 Å. Chelated lanthanide donor-enabled TR-FRET allows measurement from autofluorescent biological samples.

Here, we describe a rapid TR-FRET-based method for antigen detection and use SARS-CoV-2 NP and SP as the model antigens. In the assay, polyclonal anti-NP and anti-RBD rabbit antibodies, each labeled with either a donor or an acceptor fluorophore, are combined at an equimolar ratio and mixed with the clinical sample. The antigen, if present, binds the labeled antibodies and brings the fluorophores into proximity. This results in a TR-FRET signal upon excitation, indicating the presence of the antigen. We initially demonstrate the limits of detection (LODs) for recombinant NP and SP as well as cell culture-grown SARS-CoV-2. We then evaluate the assay performance among 48 RT-PCR-positive and 96 negative clinical NPS samples and compare the antigen detection results to those of RT-PCR and virus cultivation.

## RESULTS

### Proof of concept for the homogeneous antigen detection assay.

**FIG 1:**
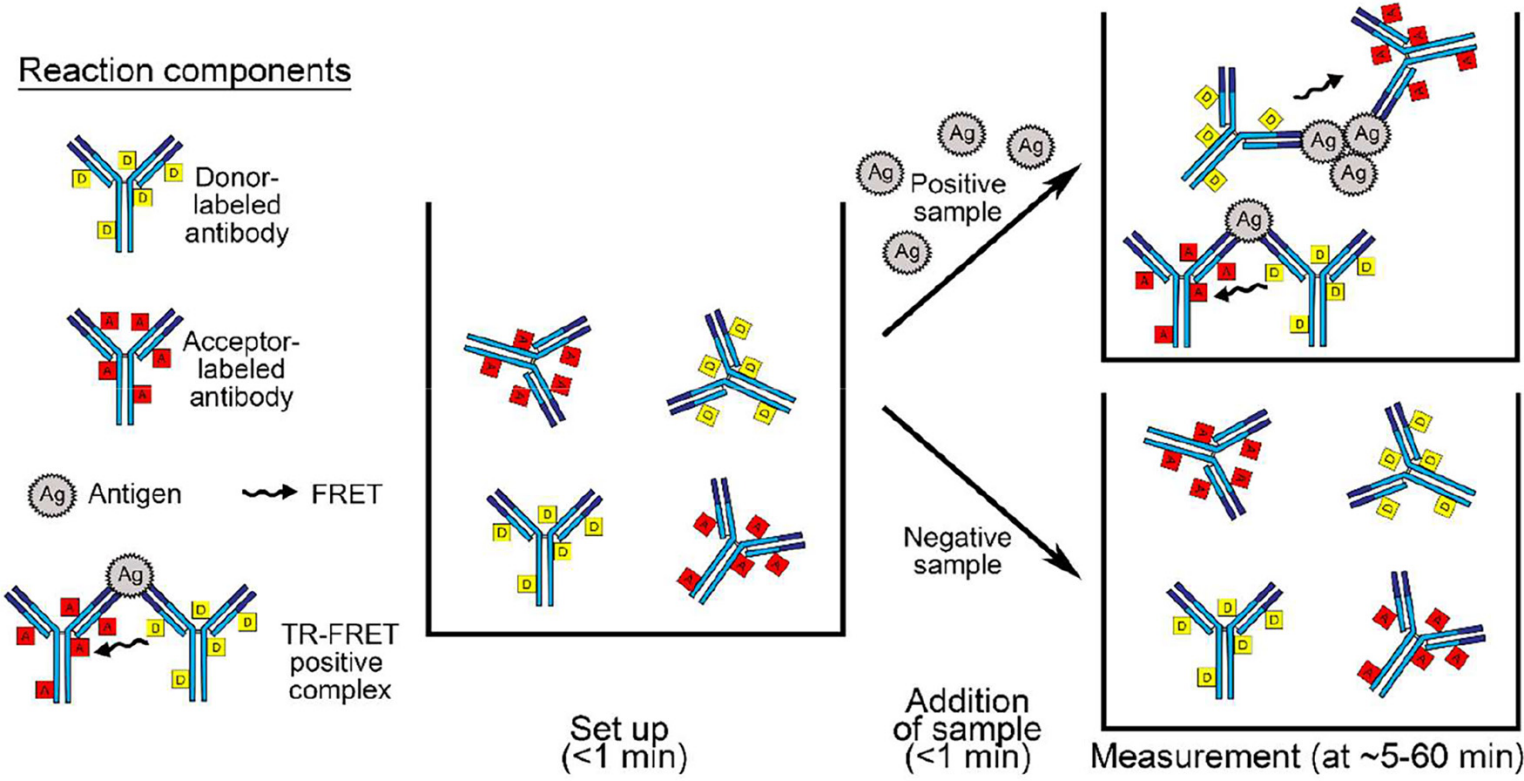
The TR-FRET assay workflow and principle. The left side shows the reaction components. At the center is a well containing donor- and acceptor-labeled antibodies at a 1:1 molar ratio in the reaction buffer; in our setup, the total antibody concentration at this point is 24 nM, and the volume is 10 μl. The arrows indicate the addition of the sample material, in our setup either 10 μl of purified recombinant protein or 10 μl of an NPS sample. The top right side shows schematically the antigen-antibody complexes formed following the addition of a sample containing the antigen; the reaction volume at this stage in our setup is 20 μl. The bottom right side schematically demonstrates that the labeled antibodies do not form TR-FRET active complexes in the absence of the antigen.

We hypothesized that homogeneous, i.e., in-solution, detection of antigens could be achieved utilizing a polyclonal antibody separately labeled with fluorophores forming a FRET pair. To test the hypothesis and the assay principle presented in Fig. 1, we generated antisera against SARS-CoV-2 NP and the RBD of SP, with titers of >204,800 based on NP and SP enzyme-linked immunosorbent assays (ELISAs), respectively. Following affinity purification, the antibodies were labeled with chelated europium (Eu) (donor) and Alexa Fluor 647 (AF647) (acceptor). In the first experiments, we tested the assay principle by mixing recombinant NP and SP with 1 μM (500 nM Eu-labeled and 500 nM AF647-labeled) anti-NP and anti-RBD antibody mixtures in the presence of increasing bovine serum albumin (BSA) concentrations. The addition of BSA increased the signal-to-noise ratio, encouraging us to evaluate the assay performances further by utilizing infectious SARS-CoV-2-containing cell culture supernatants and different buffer compositions. The assay with 1 μM antibody concentrations produced respectable signal-to-noise ratios in detergent-containing RIPA (radioimmunoprecipitation assay) buffer (see Fig. S1 in the supplemental material). The RIPA buffer (see Materials and Methods for the full recipe) used here contained 1% NP-40 and 0.1% SDS, both of which have been shown to effectively inactivate the enveloped SARS-CoV-2 (15, 16), which is why we decided to employ RIPA buffer for the subsequent analyses.

10.1128/mBio.00902-21.1 FIG S1 TR-FRET antigen assay in different buffer compositions. The antibody concentrations refer to the total amounts used in the reaction mixtures, i.e., a 1:1 mixture of Eu- and AF647-labeled antibodies. (a) Anti-NP at a 1 μM concentration. (b) Anti-NP at a 1 μM concentration. The *y* axis (and values over bars) indicates the fold increase in the HTRF ratio (HTRF_sample_/HTRF_buffer_). Mock snt, supernatant from mock-infected Vero E6 cells; SARS-CoV-2 snt-1 and -2, supernatants containing infectious SARS-CoV-2. Download FIG S1, PDF file, 0.2 MB. Copyright © 2021 Rusanen et al. 2021 Rusanen et al. https://creativecommons.org/licenses/by/4.0/ This content is distributed under the terms of the Creative Commons Attribution 4.0 International license.

### Assay optimization using recombinant antigens and SARS-CoV-2.

To optimize the assay performance, we mixed the labeled antibodies at equimolar ratios with known amounts of recombinant NP and SP and recorded the produced TR-FRET signals (as HTRF [homogeneous time-resolved fluorescence] values) as a function of time. The results showed that the assays produced the highest HTRF values when the concentration of labeled antibodies equaled the concentrations of the purified respective antigens (Fig. S2). Higher antibody concentrations shortened the time required to reach the signal peak (fold increase in the HTRF value, HTRF_sample_/HTRF_buffer_): with an antibody concentration of 5 nM (2.5 nM Eu- plus 2.5 nM AF647-labeled antibodies), it took ∼60 min for the signal to peak for both NP and SP, whereas with an antibody concentration of 500 nM, the NP signal peak occurred in 7 min, and the SP signal peaked in ∼30 min. We also tested the assay performances with Eu- and AF-labeled antibodies mixed at unequal proportions of 1:2 and 2:1, but this did not increase the signal-to-background ratio (Table S1).

10.1128/mBio.00902-21.2 FIG S2 TR-FRET cross-titration of labeled antibodies and purified recombinant nucleoprotein (NP) and spike glycoprotein (SP). (a) Anti-NP antibody concentration of 5 nM against NP concentrations of 5, 50, and 500 nM. (b) Anti-RBD antibody concentration of 5 nM against SP concentrations of 5, 50, and 500 nM. (c) Anti-NP antibody concentration of 50 nM against NP concentrations of 5, 50, and 500 nM. (d) Anti-RBD (receptor-binding domain) antibody concentration of 50 nM against SP concentrations of 5, 50, and 500 nM. (e) Anti-NP antibody concentration of 500 nM against NP concentrations of 5, 50, and 500 nM. (f) Anti-RBD antibody concentration of 500 nM against SP concentrations of 5, 50, and 500 nM. The antibody concentrations refer to the total amounts used in the reaction mixtures, i.e., a 1:1 mixture of Eu- and AF647-labeled antibodies. The *y* axis indicates the fold increase in the HTRF ratio (HTRF_sample_/HTRF_buffer_). The *x* axis shows the time in minutes since the pipetting of the samples onto the plate began (∼5 min before the first measurement). Download FIG S2, PDF file, 0.2 MB. Copyright © 2021 Rusanen et al. 2021 Rusanen et al. https://creativecommons.org/licenses/by/4.0/ This content is distributed under the terms of the Creative Commons Attribution 4.0 International license.

10.1128/mBio.00902-21.10 TABLE S1 Comparison of TR-FRET antigen assay results, expressed as HTRF ratios (HTRF_sample_/HTRF_buffer_), using different ratios of Eu- and AF647 (Alexa Fluor 647)-labeled anti-NP (nucleoprotein) and anti-RBD (receptor-binding domain) antibodies. Download Table S1, PDF file, 0.1 MB. Copyright © 2021 Rusanen et al. 2021 Rusanen et al. https://creativecommons.org/licenses/by/4.0/ This content is distributed under the terms of the Creative Commons Attribution 4.0 International license.

We then evaluated the assay performance at 50, 25, 12, and 6 nM total antibody concentrations using SARS-CoV-2-containing cell culture supernatants at different dilutions. We included a UV-inactivated cell culture supernatant to find out whether UV inactivation would affect the analysis. The results concurred with the findings using recombinant antigens and showed the dependence of the TR-FRET signal kinetics on the antigen concentration (Fig. S3). The results further indicated that a 12 nM total antibody concentration enables the measurement of antigens over a broad range of virus concentrations and that infectious and UV-inactivated SARS-CoV-2 produce similar results.

10.1128/mBio.00902-21.3 FIG S3 TR-FRET cross-titration of labeled antibodies and virus-containing cell culture supernatants. The antibody concentrations refer to the total amounts used in the reaction mixture, i.e., a 1:1 mixture of Eu- and AF647-labeled antibodies. The different antibody concentrations were titrated against a SARS-CoV-2-containing cell culture supernatant spiked at various dilutions in a pool of negative NPS samples. (a) Anti-NP antibody at 50 nM. (b) Anti-RBD antibody at 50 nM. (c) Anti-NP antibody at 25 nM. (d) Anti-RBD antibody at 25 nM. (e) Anti-NP antibody at 12 nM. (f) Anti-RBD antibody at 12 nM. (g) Anti-NP antibody at 6 nM. (h) Anti-RBD antibody at 6 nM. The *y* axis (log scale) indicates the fold increase in the HTRF ratio (HTRF_sample_/HTRF_buffer_). The *x* axis shows the time in minutes since the first measurement began. Download FIG S3, PDF file, 0.2 MB. Copyright © 2021 Rusanen et al. 2021 Rusanen et al. https://creativecommons.org/licenses/by/4.0/ This content is distributed under the terms of the Creative Commons Attribution 4.0 International license.

### Limits of detection.

To assess the limits of detection (LODs) for the assays at a 12 nM total antibody concentration, we spiked a pool of NPS samples with either purified antigens or inactivated SARS-CoV-2 at different dilutions. With purified antigens, the lowest concentrations producing readily detectable signals were 0.05 nM for NP and 0.5 nM for SP (Fig. S4a and b). With UV-inactivated SARS-CoV-2, dilutions of the supernatant up to 1:5,120 and 1:160 produced reliably measurable signals with anti-NP and anti-RBD antibodies, respectively (Fig. S4c and d). With the 10-μl sample volume, the detection limits of the assay were ∼25 pg for recombinant NP (using a molecular weight of 50 kDa) and 875 pg for SP (using a molecular weight of 175 kDa). Correspondingly, the NP assay could detect approximately 15 PFU and the RBD assay could detect approximately 420 PFU (converted by using the formula 0.7 × 50% tissue culture infectious doses [TCID_50_] per milliliter = PFU per milliliter) per reaction.

10.1128/mBio.00902-21.4 FIG S4 The limit of detection for TR-FRET antigen detection using NPS samples spiked with recombinant antigens or inactivated virus. The evaluation was done with total antibody concentrations of 12 nM, i.e., 6 nM Eu- and 6 nM AF647-labeled antibodies. (a) Recombinant nucleoprotein (NP) spiked at 0.5 fM to 5 nM in a pool of negative NPS samples. (b) Recombinant spike glycoprotein (SP) spiked at 0.5 fM to 5 nM in a pool of negative NPS samples. (c) UV-inactivated SARS-CoV-2-containing cell culture supernatant spiked at 1:20,480 to 1:10 in a negative NP swab sample, with labeled anti-N antibodies at 6 and 6 nM. (d) Inactivated SARS-CoV-2 spiked at 1:20,480 to 1:10 in a pool of negative NPS samples. The *y* axis (log scale) indicates the fold increase in the HTRF ratio (HTRF_sample_/HTRF_buffer_). The *x* axis shows the time in minutes since the first measurement began. RBD, receptor-binding domain. Download FIG S4, PDF file, 0.2 MB. Copyright © 2021 Rusanen et al. 2021 Rusanen et al. https://creativecommons.org/licenses/by/4.0/ This content is distributed under the terms of the Creative Commons Attribution 4.0 International license.

### Detection of SARS-CoV-2 antigens in NPS samples.

After setting up the assay conditions using recombinant proteins and cell culture-grown virus, we tested the assays for the detection of viral antigen in SARS-CoV-2 RT-PCR-positive NPS samples. We had 48 NPS samples with RT-PCR cycle threshold (*C_T_*) values linearly ranging from ∼12 to 30 (Fig. S5) and employed total antibody concentrations of 50, 25, 12, and 6 nM. We observed that samples with *C_T_* values of ≤25 yielded a signal in the NP assay (Fig. S6). By using the optimized conditions with 12 nM total labeled antibodies, the sensitivity of the NP TR-FRET assay in comparison with RT-PCR was 77.1% (37/48). The SP assay showed greater diversity; most samples with *C_T_* values of ≤15 yielded a positive result (Fig. S6). We also performed immunoblotting to detect NP and SP in NPS samples covering the *C_T_* value range of 12.8 to 26.2 and could detect SP and NP in samples with *C_T_* values of <22 (Fig. S7).

10.1128/mBio.00902-21.5 FIG S5 Distribution of the *C_T_* values in the original diagnostic SARS-CoV-2 RT-PCR. The *y* axis shows the *C_T_* value in the RT-PCR, and the *x* axis shows the sample number. Download FIG S5, PDF file, 0.1 MB. Copyright © 2021 Rusanen et al. 2021 Rusanen et al. https://creativecommons.org/licenses/by/4.0/ This content is distributed under the terms of the Creative Commons Attribution 4.0 International license.

10.1128/mBio.00902-21.6 FIG S6 Time-resolved Förster resonance energy transfer (TR-FRET) antigen assay results for NPS samples at various antibody concentrations (6 to 50 nM) against *C_T_* values of the positive SARS-CoV-2 RT-PCR results. (a) Anti-NP (nucleoprotein) antibody at a 6 nM concentration. (b) Anti-NP antibody at a 12 nM concentration. (c) Anti-NP antibody at a 25 nM concentration. (d) Anti-NP antibody at a 50 nM concentration. (e) Anti-RBD (receptor-binding domain) antibody at a 6 nM concentration. (f) Anti-RBD antibody at a 12 nM concentration. (g) Anti-RBD antibody at a 25 nM concentration. (h) Anti-RBD antibody at a 50 nM concentration. The *y* axis (log scale) indicates the fold increase in the HTRF ratio (HTRF_sample_/HTRF_buffer_) measured directly after pipetting of the samples onto the plate. The *x* axis shows the cycle threshold (*C_T_*) values measured in the diagnostic SARS-CoV-2 RT-PCR. The coloring in the graphs indicates the presence (red) or absence (blue) of cytopathic effect (CPE) following inoculation of VE6-TMPRSS2-H10 cells with 50 μl of the NPS sample. Eu, europium; AF, Alexa Fluor 647. Download FIG S6, PDF file, 0.3 MB. Copyright © 2021 Rusanen et al. 2021 Rusanen et al. https://creativecommons.org/licenses/by/4.0/ This content is distributed under the terms of the Creative Commons Attribution 4.0 International license.

10.1128/mBio.00902-21.7 FIG S7 Detection of SARS-CoV-2 antigens in selected NPS samples by immunoblotting. The NPS samples selected based on the *C_T_* values in the diagnostic SARS-CoV-2 RT-PCR were separated on SDS-PAGE gels (4 to 15% Mini-Protean TGX precast protein gel, deep well; Bio-Rad), at approximately 40 μl of NPS sample/well, using a standard protocol but under nonreducing conditions. The proteins were transferred (standard wet blotting protocol) onto a nitrocellulose membrane (nitrocellulose blotting membrane, Amersham Protran 0.45-μm NC; GE Healthcare) and following blocking (30 min at room temperature with blocking buffer [3% skimmed milk powder in a solution containing 50 mM Tris, 150 mM NaCl, and 0.05% Tween 20]) were first probed with anti-RBD antiserum (1:3,000 dilution in blocking buffer), and binding was detected using IRDye 800CW donkey anti-rabbit IgG secondary antibody (Li-Cor Biosciences) at a 1:10,000 dilution in blocking buffer. The membrane was then probed with anti-NP antiserum (1:4,000 dilution in blocking buffer), and the same secondary antibody was used for detection. The results were recorded using an Odyssey infrared imaging system (Li-Cor Biosciences). (a) Results of anti-RBD (detects S protein under nonreducing conditions) staining. The wells are labeled by the diagnostic SARS-CoV-2 RT-PCR values of the NPS samples. A band migrating at approximately 170 kDa (indicated by an arrow) represents the unprocessed SP, and it is detected in some of the samples. (b) Results of anti-NP staining. The labeling of wells is as described above for panel a. A band migrating at approximately 45 kDa (indicated by an arrow) represents the NP, and it is clearly detectable in samples with *C_T_* values of <21.5. Download FIG S7, PDF file, 0.4 MB. Copyright © 2021 Rusanen et al. 2021 Rusanen et al. https://creativecommons.org/licenses/by/4.0/ This content is distributed under the terms of the Creative Commons Attribution 4.0 International license.

### Association between infectious virus and antigen detection.

**FIG 2:**
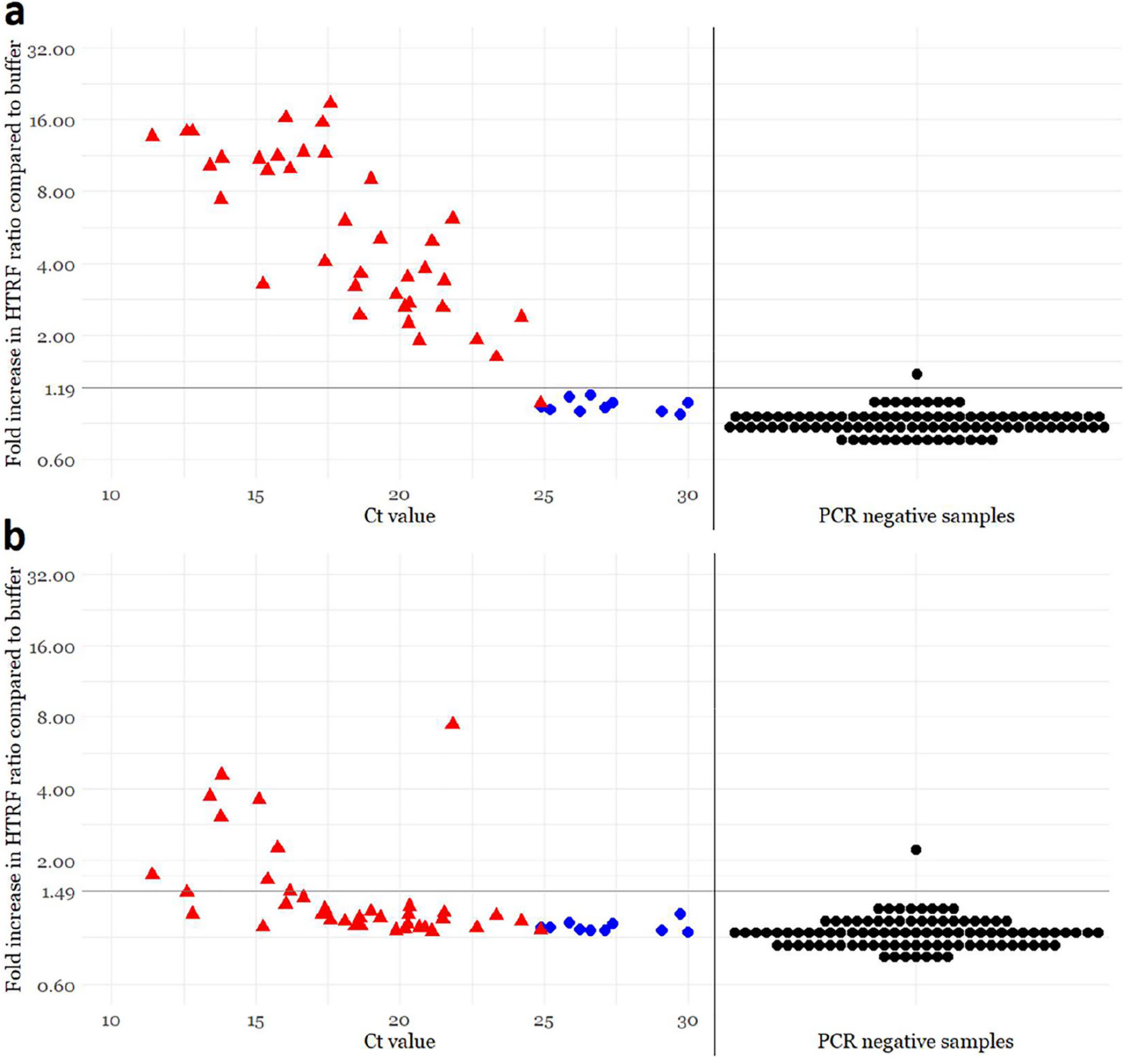
Comparison of time-resolved Förster resonance energy transfer (TR-FRET)-based antigen detection and the amount of virus as analyzed by SARS-CoV-2 RT-PCR and virus isolation from NPS samples. The total antibody concentration in the assay mixtures is 12 nM (6 nM Eu- and 6 nM AF647-labeled antibodies). (a) Anti-NP (nucleoprotein) assay results. (b) Anti-RBD (receptor-binding domain) assay results. The *y* axis (log scale) indicates the fold increase in the homogeneous time-resolved fluorescence (HTRF) ratio (HTRF_sample_/HTRF_buffer_) measured directly after pipetting the samples onto the plate. The *x* axis shows the *C_T_* values measured in the diagnostic SARS-CoV-2 RT-PCR. The horizontal black line is the antigen test positivity cutoff, corresponding to the average plus 4 standard deviations of the signals induced by SARS-CoV-2 RT-PCR-negative samples. The vertical black line separates SARS-CoV-2 RT-PCR-positive (*n* = 48) and -negative (*n* = 96) NPS samples. The coloring in the graphs indicates the presence (red) or absence (blue) of cytopathic effect (CPE) following inoculation of VE6-TMPRSS2-H10 cells with 50 μl of the NPS sample. Black, not cultured.

To determine to what extent the antigen assays correspond to the amounts of infectious virus in the sample, we subjected the 48 SARS-CoV-2 RT-PCR-positive NPS samples to virus isolation. Transmembrane serine protease 2 (TMPRSS2) has been reported to function in priming SARS-CoV-2 spike for entry (17), and we thus chose to use both wild-type Vero E6 cells and a clonal population of TMPRSS2-expressing Vero E6 cells (VE6-TMPRSS2-H10) (Fig. S8). As indicated by cytopathic effects (CPEs) as well as RT-PCR from the cell culture supernatants, SARS-CoV-2 was isolated from 35/48 and 38/48 NPS samples with Vero E6 and VE6-TMPRSS2-H10 cells, respectively. Interestingly, the cell culture supernatants from VE6-TMPRSS2-H10 cells yielded a positive result 3 to 5 cycles earlier than did the supernatants from Vero E6 cells, pointing to ∼10 to 40 times more efficient virus production (Fig. S9). Altogether, infectious SARS-CoV-2 was recovered from all samples showing *C_T_* values of ≤24.5. We then compared the antigen assay to virus isolation from the respective samples and observed that all of the samples with *C_T_* values of ≤24.5 were positive in the NP assay (Fig. 2a). Of the samples with *C_T_* values of >24.5, all produced a negative result in the TR-FRET NP assay, and SARS-CoV-2 was recovered from only one sample (*C_T_*, 24.87). By using the optimized conditions with 12 nM total labeled antibodies, the sensitivity of the NP assay in comparison with virus isolation was 97.4% (37/38). The performance of the SP assay was poorer; only 8/38 samples with recoverable SARS-CoV-2 yielded a positive result (Fig. 2b).

10.1128/mBio.00902-21.8 FIG S8 Detection of transmembrane serine protease 2 (TMPRSS2) in the clonal population of lentivirus-transduced Vero E6 cells expressing TMPRSS2 (VE6-TMPRSS2-H10). Wild-type Vero E6 and VE6-TMPRSS2-H10 cells were collected from a 6-well plate using a cell scraper, washed twice with PBS, and lysed in Laemmli sample buffer. The cell lysates were separated on an SDS-PAGE gel (4 to 20% Mini-Protean TGX precast protein gel; Bio-Rad) using standard protocols. The proteins were transferred (standard wet blotting protocol) onto a nitrocellulose membrane (nitrocellulose blotting membrane, Amersham Protran 0.45-μm NC; GE Healthcare) and following blocking (30 min at room temperature in blocking buffer [3% skimmed milk powder in a solution containing 50 mM Tris, 150 mM NaCl, and 0.05% Tween 20]) were probed with mouse monoclonal anti-V5 epitope tag antibody (clone E10/V4RR; Invitrogen) diluted 1:2,000 in blocking buffer. Binding was detected using IRDye 800CW donkey anti-mouse IgG secondary antibody (Li-Cor Biosciences) at a 1:10,000 dilution in blocking buffer, and the results were recorded using an Odyssey infrared imaging system (Li-Cor Biosciences). The band indicated by an arrow at approximately 60 to 65 kDa by migration indicates the expression of V5-tagged TMPRSS2 by the VE6-TMPRSS2-H10 clone of Vero E6 cells. Download FIG S8, PDF file, 0.2 MB. Copyright © 2021 Rusanen et al. 2021 Rusanen et al. https://creativecommons.org/licenses/by/4.0/ This content is distributed under the terms of the Creative Commons Attribution 4.0 International license.

10.1128/mBio.00902-21.9 FIG S9 RT-PCR results from supernatants collected at 5 days postinoculation with SARS-CoV-2 RT-PCR-positive NPS samples. The *y* axis shows *C_T_* values from SARS-CoV-2 RT-PCR from the cell culture supernatant, and the *x* axis shows the respective *C_T_* values of the original diagnostic test. The blue dots indicate the supernatant from an isolation attempt on Vero E6 cells, and the orange dots indicate the supernatant from an isolation attempt on VE6-TMRPRSS2-H10 cells. Download FIG S9, PDF file, 0.1 MB. Copyright © 2021 Rusanen et al. 2021 Rusanen et al. https://creativecommons.org/licenses/by/4.0/ This content is distributed under the terms of the Creative Commons Attribution 4.0 International license.

### False positivity and cross-reactivity.

**FIG 3:**
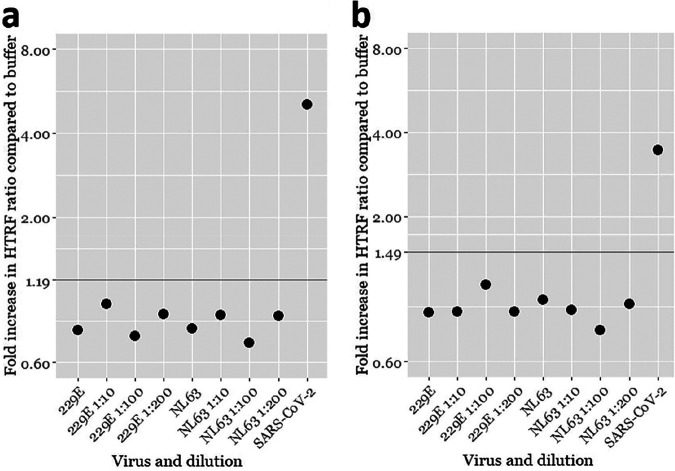
SARS-CoV-2 TR-FRET antigen assay cross-reactivity evaluated with cell culture supernatants of seasonal human coronaviruses hCoV-229E and -NL63. (a) Anti-NP assay results with hCoV-229E and -NL63 cell culture supernatants at different dilutions. (b) Anti-NP assay results with hCoV-229E and -NL63 cell culture supernatants at different dilutions. The *y* axis (log scale) indicates the fold increase in the HTRF ratio (HTRF_sample_/HTRF_buffer_). The horizontal lines indicate the respective TR-FRET assay cutoffs. A UV-inactivated SARS-CoV-2-containing cell culture supernatant is included as a positive control.

After finding a total antibody concentration of 12 nM ideal for the assay performance, we were interested in knowing the rate of false-positive results. To that end, we tested 96 SARS-CoV-2 RT-PCR-negative NPS samples in the TR-FRET antigen assays. In parallel, we studied the potential cross-reactivity of the cell culture-grown seasonal common cold coronaviruses hCoV-229E (human coronavirus 229E) and hCoV-NL63 in the TR-FRET assays. Among the SARS-CoV-2 RT-PCR-negative NPS samples, only one produced a positive HTRF signal. We reanalyzed this sample with another RT-PCR assay (Xpert Xpress SARS-CoV-2; Cepheid), confirming the negative result. To assess the antigen specificity of the signal, we also analyzed this sample using mismatching combinations of the labeled anti-NP and anti-RBD antibodies. All of the combinations yielded a positive result, suggesting that something other than the antigens brings the labeled antibodies together. By using the optimized conditions with 12 nM total labeled antibodies, the specificities of NP and SP TR-FRET assays in comparison with RT-PCR were 99.0% (95/96), and those in comparison with virus isolation were 100% (10/10). We then set as cutoffs for the TR-FRET assays the average plus 4 standard deviations of the signals from the SARS-CoV-2 RT-PCR-negative NPS samples (excluding the single outlier). Using these cutoffs, neither hCoV-229E nor hCoV-NL63 yielded a signal above the respective cutoffs in the NP or SP TR-FRET assay (Fig. 3). We were unable to obtain other hCoVs (OC43 and HKU1) as virus isolates or as NPS samples in a suitable buffer and thus could not evaluate the cross-reactivity further. The results with the cutoff values selected using the negative NPS samples concurred with those by the arbitrary cutoffs utilized as described above and are summarized in Fig. 2.

## DISCUSSION

The SARS-CoV-2 epidemic that began in China in late 2019 quickly evolved into a pandemic in the spring of 2020. After an initial lag in ramping up the testing capacity, RT-PCR quickly became the gold standard of acute SARS-CoV-2 diagnostics. While RT-PCR is very sensitive in picking up individuals with acute infection, the downside is that patients recovering from COVID-19 can remain RT-PCR positive over a long period. Antigen testing, on the other hand, is somewhat less sensitive for detecting patients with acute infection; however, there appears to be a better correlation between the presence of antigen and infectious virus in the NPS samples. In this article, we describe a technique for rapid antigen detection and used SARS-CoV-2 as the model pathogen. We evaluated the test by detecting SARS-CoV-2 antigen in NPS samples and compared the test’s performance against virus isolation and RT-PCR. The assay is quick and very easy to use; furthermore, the assay is rather uncomplicated to set up provided that specific antibodies against the structural proteins of the virus are available. We employed polyclonal instead of monoclonal antibodies in the assay because we think that an assay relying on polyclonal antibodies would perform better in the case of virus variants. We think that the assay can be set up using monoclonal antibodies and likely combinations or pools of monoclonal antibodies. The fluorophores (chelated Eu and Alexa Fluor 647) are readily available, and the results can be read on any microplate reader capable of measuring time-resolved fluorescence. Notably, we set up the assay in detergent-containing RIPA buffer, which contains 1% NP-40 and 0.1% SDS, both of which inactivate SARS-CoV-2 (15, 16). Thus, collecting the NPS samples directly into this matrix would significantly increase safety for the end user.

We set up the assay for the detection of both NP and SP of SARS-CoV-2 but observed a clear difference between the LODs of the two assays, with NP being detected at an ∼35-times-lower concentration. This is likely explained by the fact that we employed an antibody directed against the RBD, which constitutes only about one-sixth of the SP. The fact that the antibody in use recognizes only a single domain could make it sterically impossible for two antibody molecules to bind a single SP molecule, and thus, we speculate that the obtained signal came from SP trimers, i.e., spikes. NP is more abundant in both virions and infected cells (see Fig. S7 in the supplemental material), which additionally contributed to the higher sensitivity of NP detection in cell culture supernatants and NPS samples. Also, the use of polyclonal antibodies theoretically enables the simultaneous binding of several antibody molecules to a single antigen molecule, thus increasing the assay sensitivity.

Performance analysis of the antigen assay using NPS samples from 48 SARS-CoV-2 RT-PCR-positive and 96 negative individuals revealed that the NP assay correctly identified 37 of the positive samples and all but 1 of the negative samples. All 37 true-positive samples had a *C_T_* value of <25 cycles in the diagnostic RT-PCR. Similar to other studies, we observed a strong association between the sample infectivity and positive antigen test results: of the 38 samples that yielded an isolate, 37 produced a positive result in the NP assay. We used 50 μl for virus isolation, while the antigen assay takes only 10 μl, which could explain why one of the samples with infectious virus was not picked up. We intentionally selected NPS samples over a broad range of *C_T_* values in SARS-CoV-2 RT-PCR to obtain an estimate of the detection limit compared to RT-PCR. The fact that we analyzed samples that were not collected fresh and had been subjected to at least one freeze-thaw cycle may have negatively affected the assay sensitivity. In any case, our test could detect 97.4% of the NPS samples with infectious virus.

Because both NP and SP antigen assays gave a positive result for a single SARS-CoV-2 RT-PCR-negative sample, we reanalyzed it using a different RT-PCR with a negative result. We also attempted virus isolation from the sample but without success. It is possible that the false-positive result is a result of cross-reactivity to a human coronavirus (hCoV) infection. Of the hCoVs, only hCoV-NL63 and SARS-CoV use the same receptor as SARS-CoV-2, i.e., angiotensin-converting enzyme 2 (ACE2) (18, 19). Thus, it would be most logical that the reactivity of this sample would be due to hCoV-NL63 because both SP (based on the RBD) and NP assays gave a positive result. However, we tested the two assays using cell culture-grown hCoV-229E and hCoV-NL63 but with negative results. Unfortunately, we did not have sample material available for evaluating the potential cross-reactivity against hCoV-OC43 and hCoV-HKU1. It appears that the sample yielding a false-positive reaction contained an interfering substance, which caused the immunoglobulins to aggregate, because labeled antibodies against different antigens also yielded a TR-FRET signal. The use of mismatching antibodies could in the future serve to discern true- and false-positive results in the TR-FRET assay.

In conclusion, we describe an antigen detection assay in which the presence of an antigen is “sensed” by the simultaneous binding of two or more fluorophore-labeled antibody molecules to the antigen. We provide a proof of concept for the assay principle utilizing SARS-CoV-2 NP and SP as the model antigens and demonstrate the assay functionality utilizing patient samples. The TR-FRET-based antigen test is rapid to perform, and its results correlate well with the presence of infectious virus in clinical samples. The assay is easy to set up if a suitable antibody against SARS-CoV-2 NP and a plate reader enabling TR-FRET measurement are available. The assay sensitivity is likely lower than those of enzyme-based assays; however, the good correlation of the positive results and virus isolation could indicate that the sensitivity reached was high enough because a test with higher sensitivity would likely also pick up samples from which virus cannot be isolated. Although the assay is not as simple and robust to use as, e.g., lateral flow tests, the assay’s clear advantage is in its throughput. We estimate that a single plate reader and an experienced technician could manually analyze hundreds of specimens per hour, ideally with a 30-min turnaround time from sample arrival to results. The assay throughput could significantly be upscaled by using automation, and like RT-PCR, sample collection would represent the major limiting factor. NPS sampling directly into a detergent-containing buffer increases the assay’s user safety. We envision that the assay could be applied widely in the field, e.g., hospitals, retirement homes, airports, train stations, and schools, to identify people likely to spread the virus.

## MATERIALS AND METHODS

### Patient samples and reference results.

The evaluation of the SARS-CoV-2 TR-FRET assay was conducted by using nasopharyngeal swab (NPS) specimens collected in saline. The specimens were retrieved from patients with clinically suspected COVID-19, and they were originally sent to the HUS Diagnostic Center, HUSLAB, for SARS-CoV-2 RT-PCR testing. The specimens were subsequently stored at −20°C.

The SARS-CoV-2 RT-PCR was based on a laboratory-developed test (LDT). The details and performance of the test in our laboratory setting have been described previously (20). In this method (based on the N gene [modified from the method in reference 21]), the specimens were inactivated by combining 250 μl of MagNA Pure lysis/binding buffer (Roche Diagnostics GmbH, Mannheim, Germany) and 250 μl of the specimen. Nucleic acid extraction was done from 450 μl of the specimen lysate with the MagNA Pure viral NA SV 2.0 kit (Roche Diagnostics GmbH, Mannheim, Germany). RT-PCR was performed using the SuperScript III Platinum one-step qRT-PCR kit with 600 nM the forward primer CACATTGGCACCCGCAATC, 800 nM the reverse primer GAGGAACGAGAAGAGGCTTG, and 200 nM the probe FAM (6-carboxyfluorescein)-ACTTCCTCAAGGAACAACATTGCCA-BBQ (blackberry quencher).

The SARS-CoV-2 RT-PCR-positive panel comprised 48 specimens with cycle threshold (*C_T_*) values ranging linearly between 11.42 and 29.98 in the LDT. The SARS-CoV-2 RT-PCR-negative panel comprised 96 samples negative in the LDT. Patient data were collected and samples were handled according to a research permit approved by the local review board, permit HUS/32/2018 (Helsinki University Hospital, Finland).

### Cell lines, virus isolation, and propagation.

Vero E6 cells were transduced with a lentiviral vector expressing human transmembrane serine protease 2 (TMPRSS2) transcript variant 2 cDNA (GenBank accession number NM_005656.4) and blasticidin as a selection marker. Specifically, 1 ml of the 0.22-μm-filtered (Millipore) infectious supernatant of HEK293T cells transfected on a 10-cm plate 48 h earlier using 30 μg polyethylenimine with 5 μg pLenti6.3/V5-DEST TMPRSS2 (obtained from the Biomedicum Functional Genomics Unit, University of Helsinki), 5 μg p8.9NDSB (22), and 2 to 5 μg pMD2.G (a gift from Didier Trono [Addgene plasmid 12259 {https://n2t.net/addgene:12259}; RRID, Addgene_12259]) was added to Vero E6 cells seeded onto 6-well plates. Following 2 days of selection with 15 μg/ml of blasticidin S HCl (Invitrogen), the cells were allowed to expand until confluence and subcultured three times. Once confirmed p24 negative, a clonal population of Vero E6-TMPRSS2 cells was obtained by limiting dilution. The obtained clones (*n* = 5) were analyzed for TMPRSS2 expression by immunoblotting with V5 antibody (Invitrogen). The clone expressing the largest amount of TMPRSS2, VE6-TMPRSS2-H10, was selected for use.

SARS-CoV-2 isolation from clinical samples (stored at −20°C since the day of collection and not subjected to freeze-thawing) was attempted on both Vero E6 and VE6-TMPRSS2-H10 cells. Both cell lines were cultivated in Eagle minimal essential medium (MEM; Sigma) supplemented with 10% fetal bovine serum (FBS; Gibco), 100 IU penicillin plus 100 μg/ml streptomycin (Sigma), and 2 mM l-glutamine (Sigma). For isolation, the cells were grown on 12-well plates until approximately 90% confluent, and the growth medium was replaced with 400 μl of MEM-2% (MEM as described above but with 2% FBS), followed by the addition of 50 μl of the NPS sample (under biosafety level 3 [BSL-3] conditions) and 1 h of incubation at 37°C with 5% CO_2_. After two washes with MEM-2%, cultures were kept in 1 ml fresh MEM-2% for 4 days at 37°C (5% CO_2_), the medium was collected and clarified by centrifugation (3,000 relative centrifugal force [rcf] for 5 min), and the cells were fixed for 15 min at room temperature with 3.7% formaldehyde in phosphate-buffered saline (PBS) followed by a PBS wash and UV inactivation (500,000 μJ/cm^2^, UV Crosslinker CL-1000; Jena Analytik). The fixed cells were crystal violet stained, and the extent of cytopathic effect (CPE) was scored from 0 to 3 (from nonobservable to extensive cell death). To confirm infection, RNA was extracted (Qiagen QIAamp viral RNA extraction kit, according to the manufacturer’s protocol) from 100 μl of each cell culture supernatant, and the presence or absence of SARS-CoV-2 was analyzed by RT-PCR targeting the RdRp (RNA-dependent RNA polymerase) gene as described previously (21).

For TR-FRET antigen detection experiments, we produced a stock of SARS-CoV-2 in Vero E6 cells (23). Briefly, 90 to 95% confluent Vero E6 cells were inoculated with 500 μl of the 1:100-diluted SARS-CoV-2-containing supernatant (passage 7, approximately 5 × 10^7^ 50% tissue culture infectious doses [TCID_50_] per ml). After 1 h of virus adsorption, the medium was replaced with MEM-2%, and after 2 days at 37°C with 5% CO_2_, the supernatant was collected, clarified by centrifugation (3,000 rcf for 5 min), and stored in aliquots at −80°C. UV inactivation of the culture supernatants was done as described above.

Human coronaviruses 229E (hCoV-229E) (kindly provided by Sisko Tauriainen, University of Turku, Turku, Finland) and NL63 (hCoV-NL63) (kindly provided by Lia van der Hoek, Academic Medical Center, Amsterdam, Netherlands) served as controls for estimating the cross-reactivity of the assay. The hCoV-229E stock was produced by inoculating LLC-MK2 rhesus macaque kidney cells (from the ATCC) with 500 μl of the 1:1,000-diluted cell culture supernatant (approximately 5 × 10^9^ TCID_50_/ml) for 1 h at 37°C with 5% CO_2_. After virus adsorption, the medium was changed into MEM-2%, the cells were grown for 5 days (37°C with 5% CO_2_), and the supernatant was collected, centrifuged (3,000 rcf for 5 min), and stored in aliquots at −80°C. The hCoV-NL63 stock was produced by inoculating human lung fibroblasts (MRC-5; ATCC) with 500 μl of 1:100-diluted cell culture supernatants (approximately 1 × 10^6^ TCID_50_/ml) for 1 h at 37°C with 5% CO_2_. After virus adsorption, the media were replaced with MEM-2%, the cells were grown for 7 days (until the appearance of definitive CPE), and the supernatants were collected, centrifuged (3,000 rcf for 5 min), and stored in aliquots at −80°C. The hCoV-229E and hCoV-NL63 supernatants were inactivated for the experiments by mixing at a 1:10 dilution in RIPA buffer (50 mM Tris-HCl [pH 8.0], 150 mM NaCl, 1% NP-40, 0.1% SDS, 0.5% sodium deoxycholate, and Roche cOmplete EDTA-free protease inhibitor cocktail).

### Antigens and antibodies.

The production and purification of SARS-CoV-2 NP and SP antigens were performed according to previously described protocols (14, 24, 25). The RBD of the SP was produced in Expi293F cells as previously described (24, 25). Rabbit antisera against the RBD and NP were generated at BioGenes GmbH (Berlin, Germany) as follows: day 0 initial dose of 150 μg, day 7 booster of 75 μg, day 14 booster of 75 μg, day 28 booster of 150 μg, and day 42 final bleed. For affinity purification, the RBD and NP were coupled to CNBr-Sepharose 4B (Cytiva) according to the manufacturer’s protocol. The respective antisera were passed through coupled Sepharoses packed into Poly-Prep chromatography columns (Bio-Rad), washed with 20 column volumes of PBS, eluted (0.1 M glycine, 150 mM NaCl [pH 2.5]) with 2 M Tris (pH 9.0), concentrated using an Amicon Ultra 15-ml 100-kDa-NMWL (nominal molecular weight limit) centrifugal filter (Millipore/Merck), and dialyzed against PBS using Slide-A-Lyzer dialysis cassettes (Thermo Scientific).

### Labeling.

We labeled the affinity-purified antibodies, at 250 μg/reaction, with the donor (europium [Eu]) and acceptor (Alexa Fluor 647 [AF647]) using a QuickAllAssay Eu-chelated protein labeling kit (BN Products and Services Oy) and Alexa Fluor 647 NHS (*N*-hydroxysuccinimide) ester (Thermo Scientific) according to the manufacturer’s instructions. A disposable PD-10 desalting column with Sephadex G-25 resin (Cytiva) served to remove nonreacted fluorophores, and an Amicon Ultra 0.5-ml 50-kDa-NMWL centrifugal filter (Millipore/Merck) was used for concentrating the labeled antibodies, which were then stored aliquoted at −80°C until use.

### TR-FRET assays.

First, we set up TR-FRET assays for SARS-CoV-2 SP and NP antigens by using the respective purified proteins as well as the corresponding Eu- and AF-labeled anti-RBD and anti-NP antibodies. The assay principle and workflow are depicted in Fig. 1. Briefly, antibody mixes with equimolar concentrations of Eu- and AF-labeled anti-RBD and anti-NP antibody concentrations were prepared in RIPA buffer. For setting up the assay, a pool of four SARS-CoV-2-negative NPS samples was divided into aliquots and spiked with either NP or SP proteins at various concentrations. Ten microliters of the antibody mix was pipetted on a 384-well microplate (ProxiPlate 384 Plus F, black 384-shallow-well microplate; PerkinElmer, USA), followed by 10 μl of the antigen-spiked sample. The TR-FRET signal was measured directly thereafter and 7, 15, 22, 30, 45, 60, and 90 min after the first measurement with a Hidex Sense microplate reader (Hidex Oy, Finland). FRET donor excitation was done at 330 nm, and after a delay of 70 μs, the donor and acceptor signals were measured for 100 μs at 616 and 665 nm, respectively. TR-FRET signals were expressed as HTRF ratios, calculated as follows: HTRF ratio = emission at 616 nm/emission at 665 nm × 10,000. Thereafter, the HTRF ratios measured from the antigen-spiked samples were compared with those measured from a nonspiked sample in the same run to calculate the fold increase in the HTRF ratio. Antibody plate concentrations ranging from 5 to 500 nM (one-half Eu and one-half AF labeled) were cross-titrated with antigen plate concentrations ranging from 5 nM to 500 nM.

The ranges of antigen concentrations detectable by TR-FRET (at Eu- and AF-labeled anti-NP/RBD concentrations of 6 and 6 nM) were then assessed by performing the assay as described above using N and S plate concentrations of 5 fM to 5 nM.

To assess the assay performance with samples containing virions, cell culture supernatants containing roughly 10^7^ TCID_50_/ml of SARS-CoV-2 were used. For initial experiments (carried out in a BSL-3 laboratory), an infectious cell culture supernatant (undiluted and 1:10, 1:25, 1:50, and 1:100 diluted in RIPA buffer) was used. After verifying that UV-inactivated virus produced similar results, the negative NPS sample matrix was spiked with a UV-inactivated virus-containing cell culture supernatant to yield a dilution series from 1:10 to 1:20,480. The samples were tested in the TR-FRET assays performed as described above at Eu- and AF-labeled anti-NP/RBD concentrations of 6 and 6 nM.

NPS sample analysis was done by mixing 10 μl of the sample with 10 μl of the antibody mixes (Eu- and AF-labeled anti-NP/RBD concentrations of 6 and 6 nM). The TR-FRET assays with SARS-CoV-2 RT-PCR-positive NPS samples were carried out in a BSL-3 laboratory, and those with RT-PCR-negative samples were carried out in a BSL-2 laboratory. The signals produced by hCoV-229E and hCoV-NL63 were evaluated by mixing 10 μl (undiluted and 1:10, 1:25, 1:50, and 1:100 diluted in RIPA buffer) of the cell culture supernatant with 10 μl of the antibody mixes (at Eu- and AF-labeled anti-NP/RBD concentrations of 6 and 6 nM).
