# Supplementary figures and images for: A Generic, Scalable, and Rapid Time-Resolved Förster Resonance Energy Transfer-Based Assay for Antigen Detection—SARS-CoV-2 as a Proof of Concept
Source: mBio. 2021 May 18;12(3):e00902-21. doi: 10.1128/mBio.00902-21 (PMC8262888; doi:10.1128/mBio.00902-21)

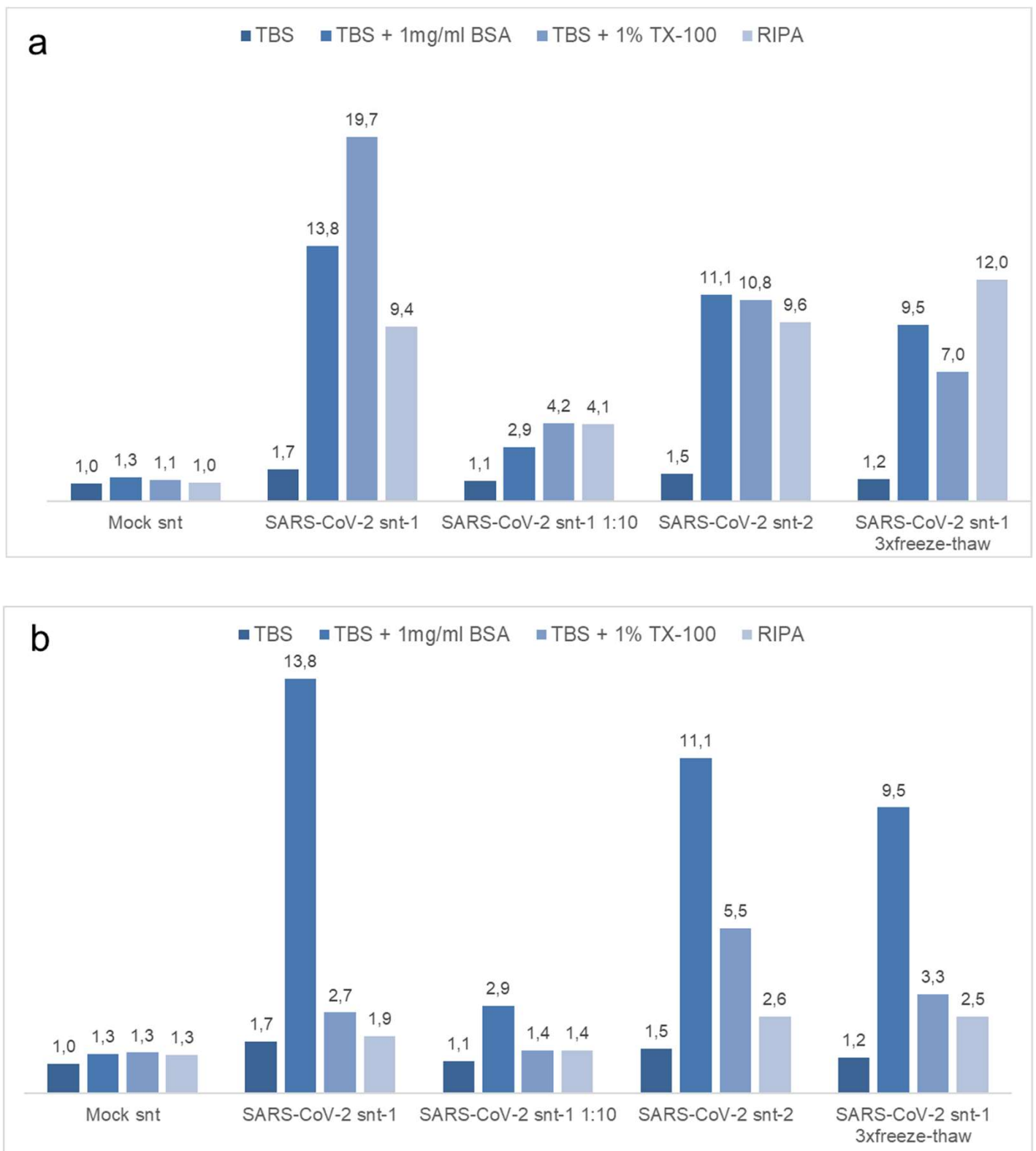

**Figure S1.**

Supplement: FIG S1 [file mbio.00902-21-sf001.pdf]

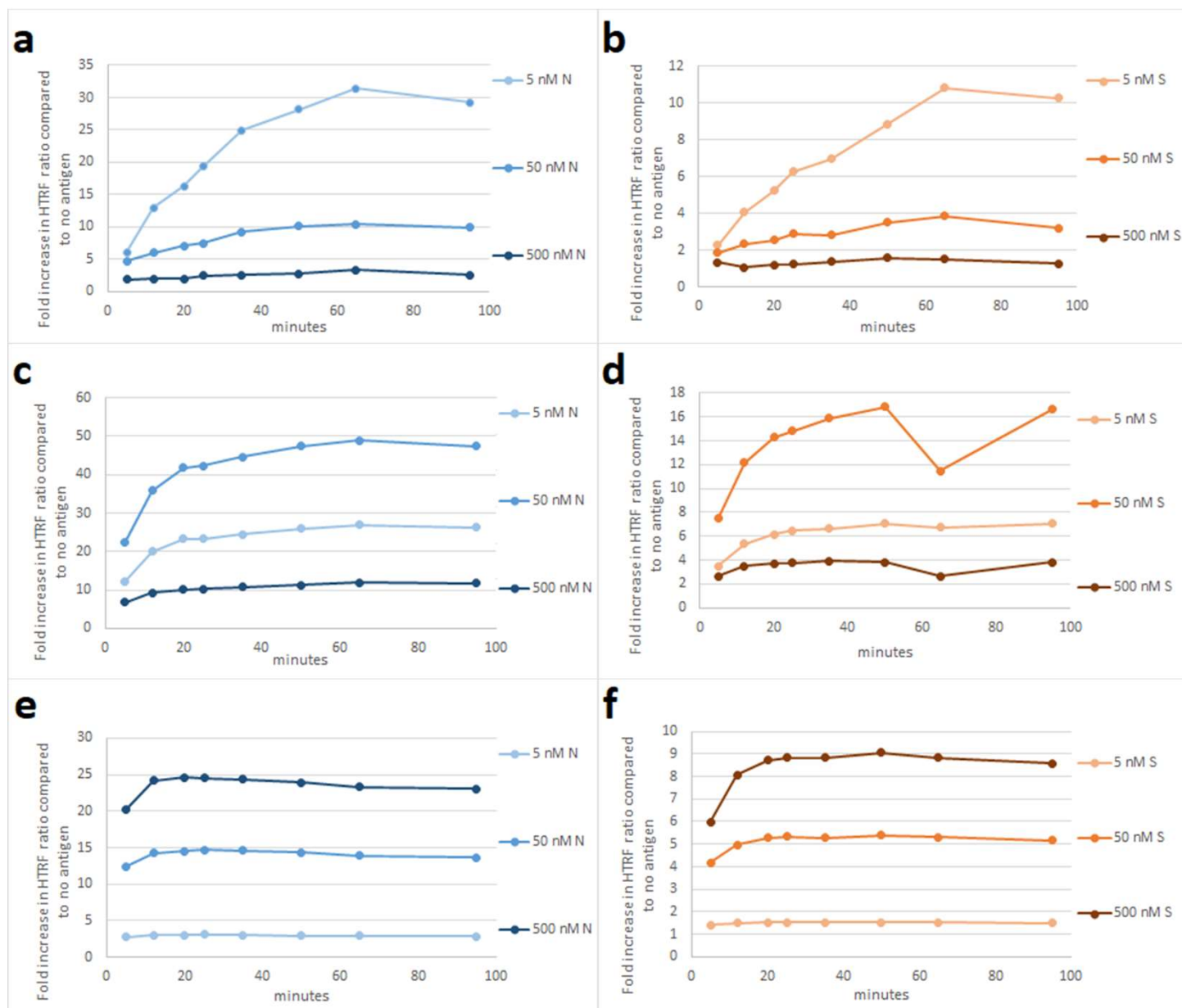

**Figure S2.**

Supplement: FIG S2 [file mbio.00902-21-sf002.pdf]

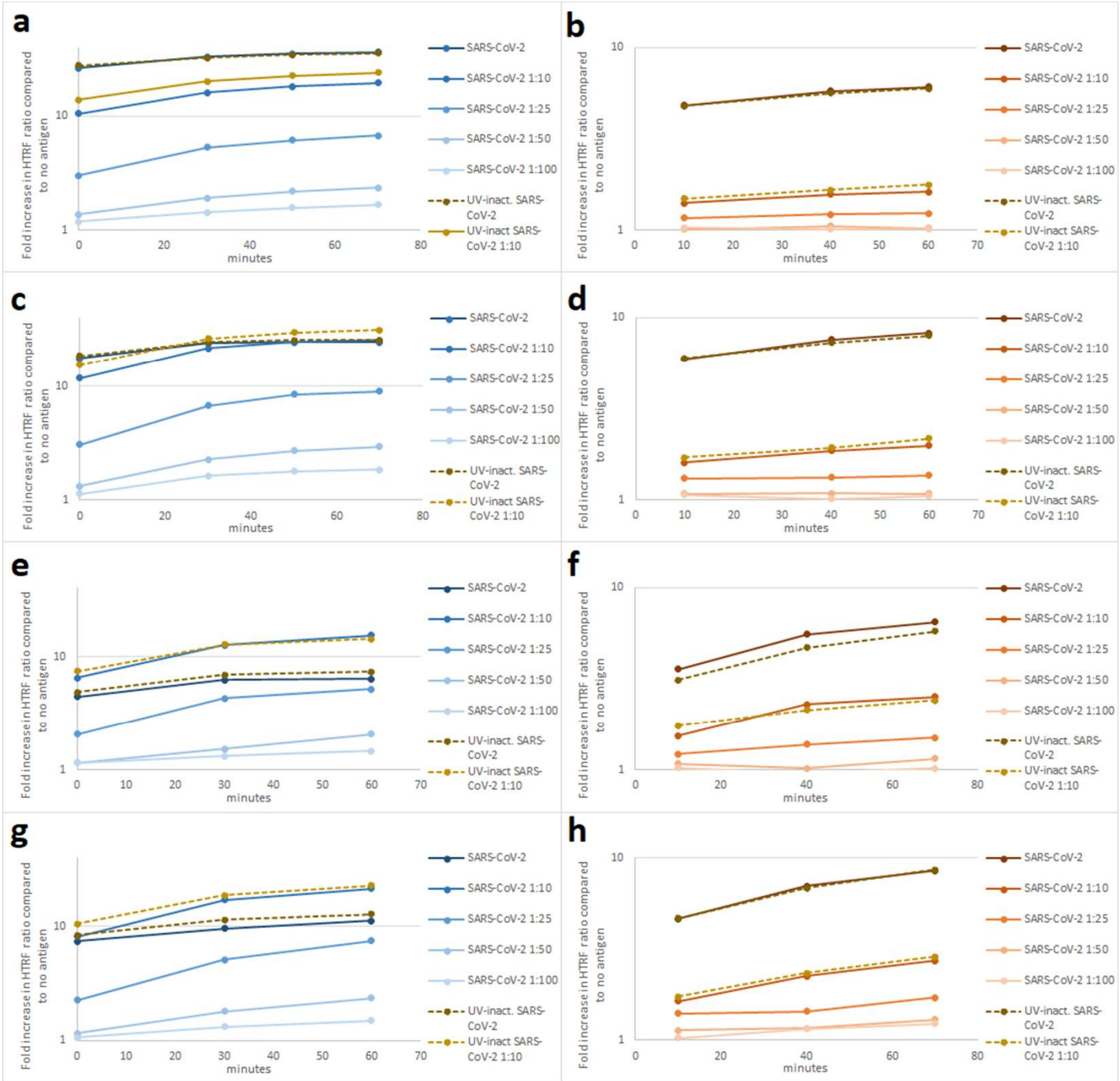

**Figure S3.**

Supplement: FIG S3 [file mbio.00902-21-sf003.pdf]

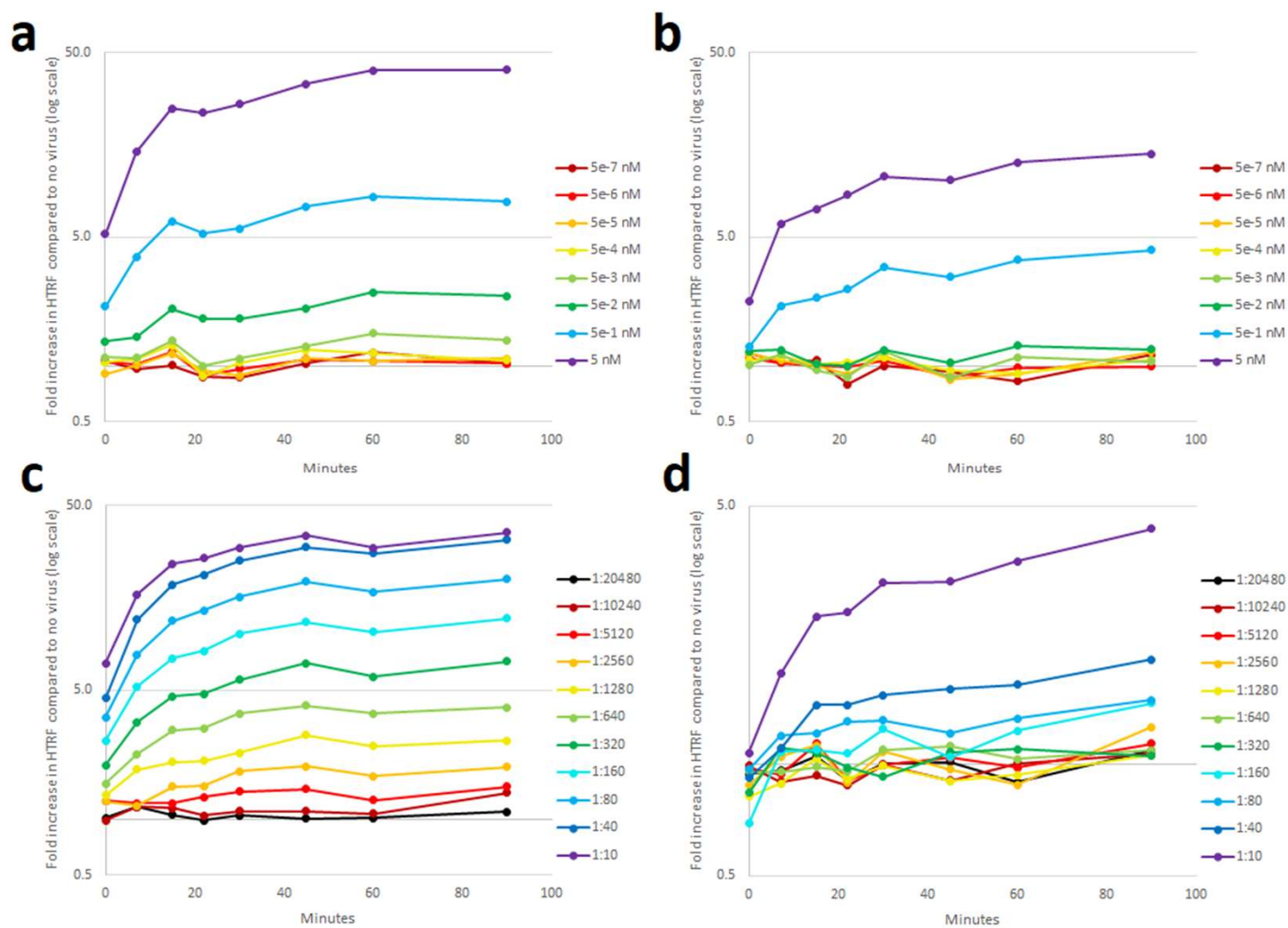

**Figure S4.**

Supplement: FIG S4 [file mbio.00902-21-sf004.pdf]

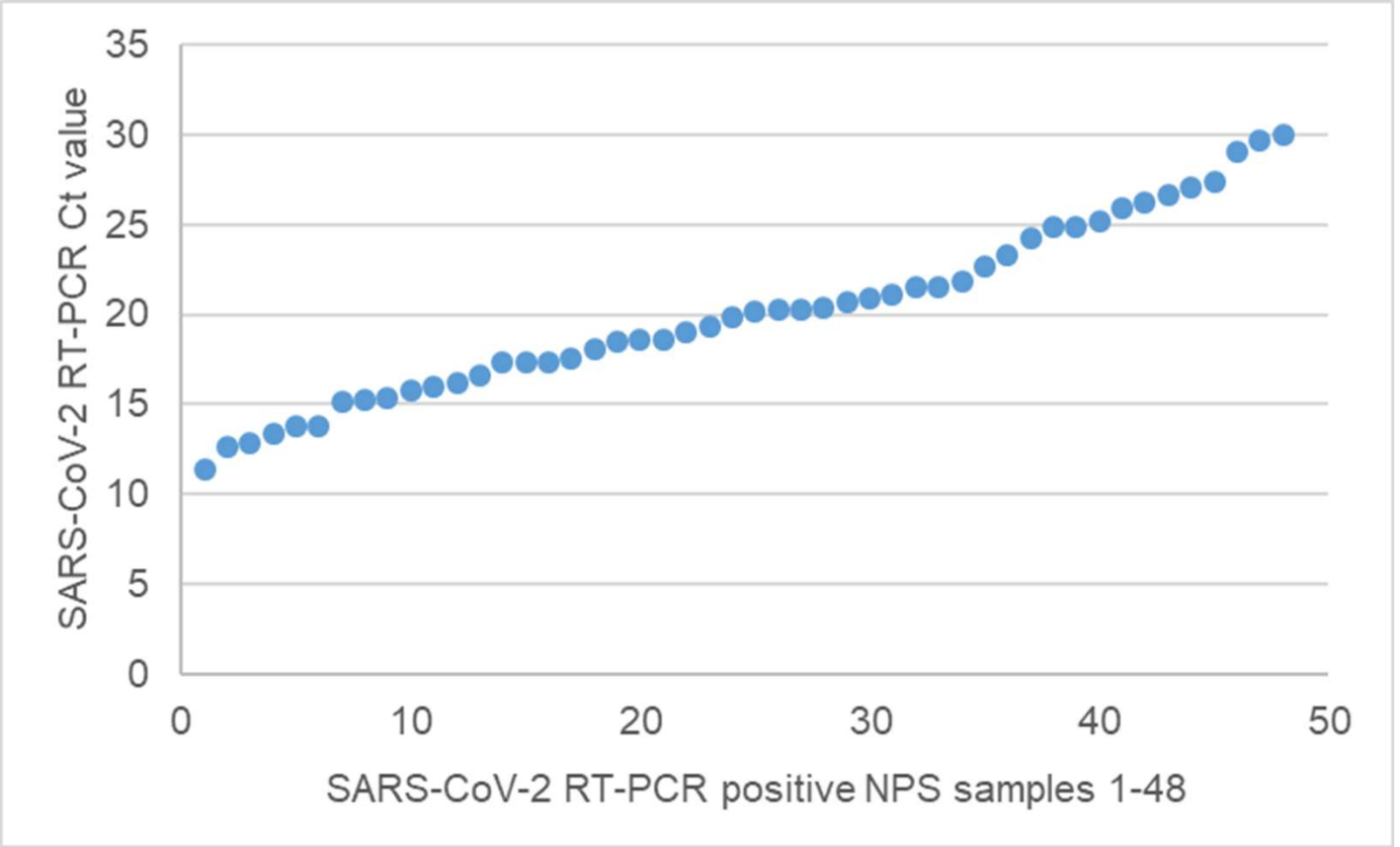

**Figure S5.**

Supplement: FIG S5 [file mbio.00902-21-sf005.pdf]

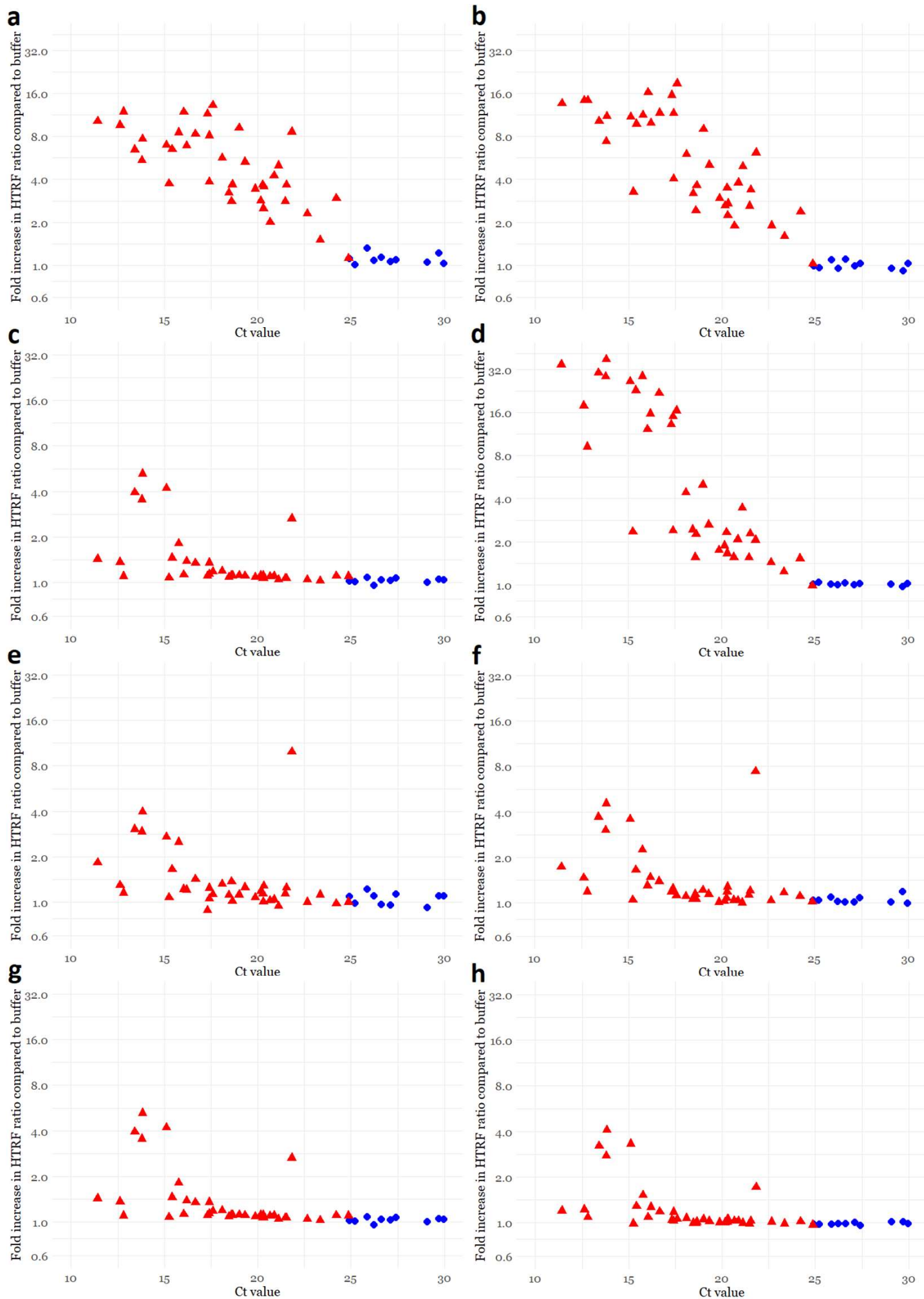

**Figure S6.**

Supplement: FIG S6 [file mbio.00902-21-sf006.pdf]

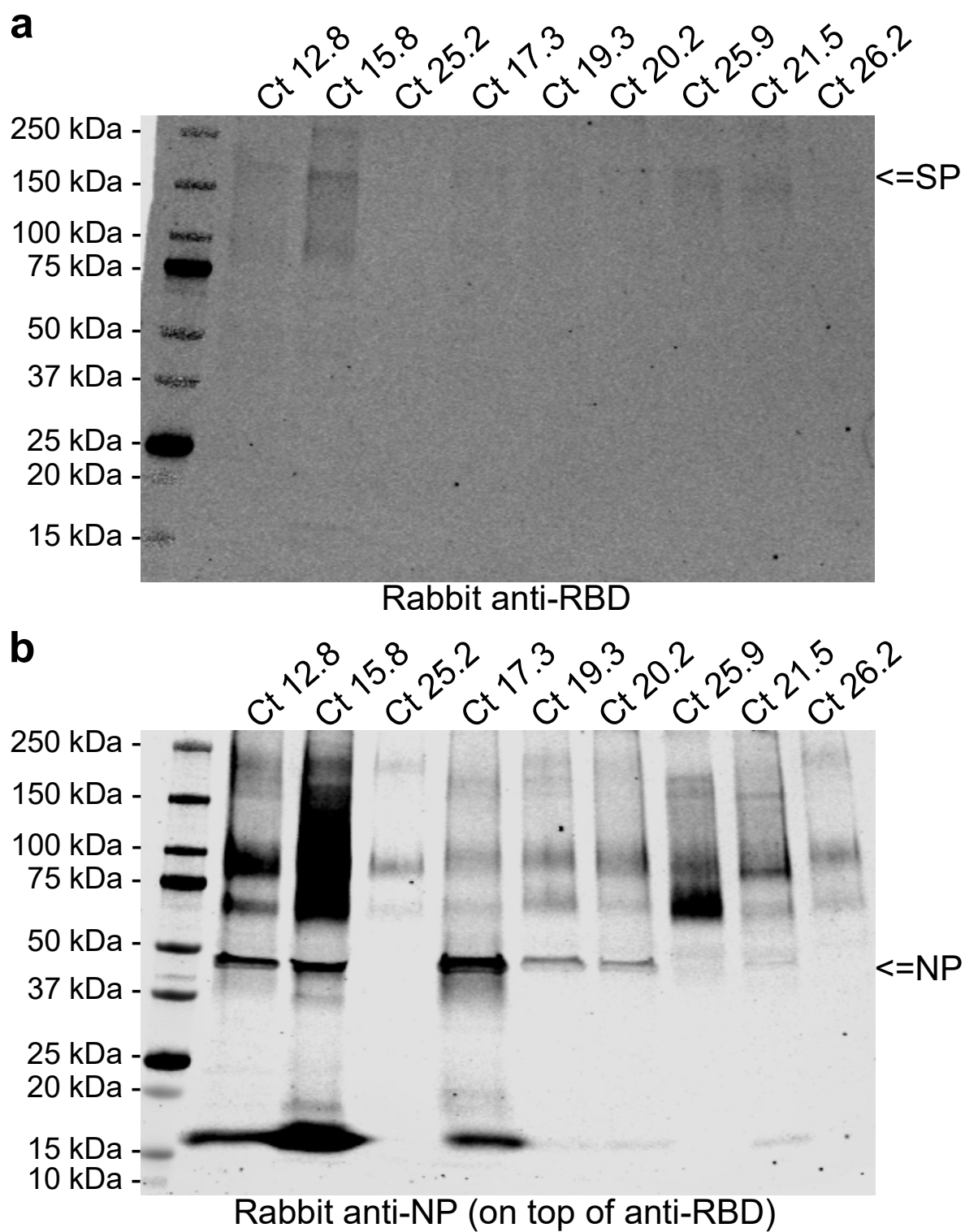

**Figure S7.**

Supplement: FIG S7 [file mbio.00902-21-sf007.pdf]

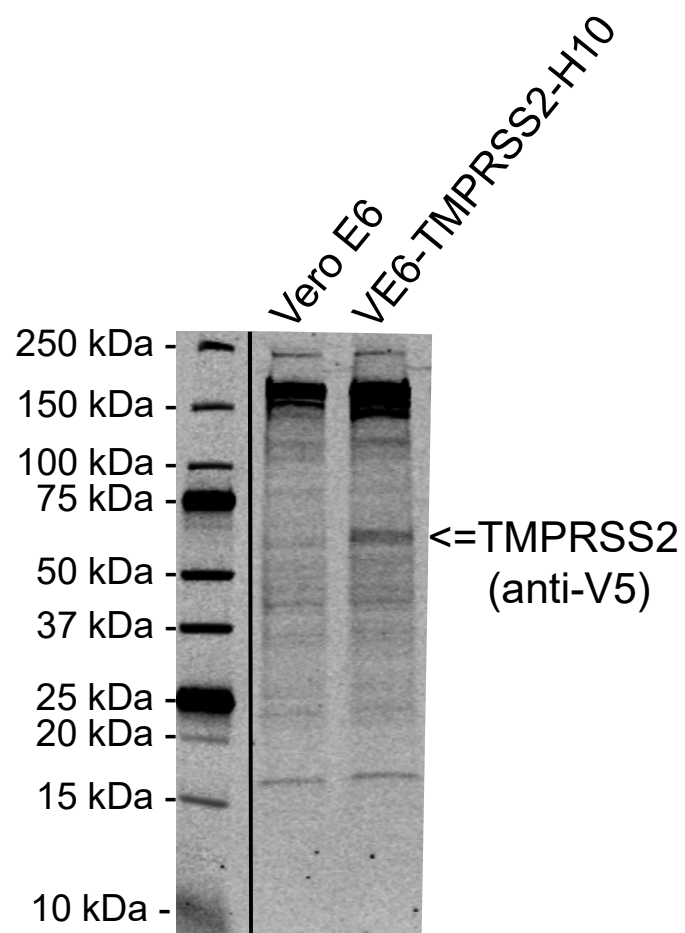

**Figure S8.**

Supplement: FIG S8 [file mbio.00902-21-sf008.pdf]

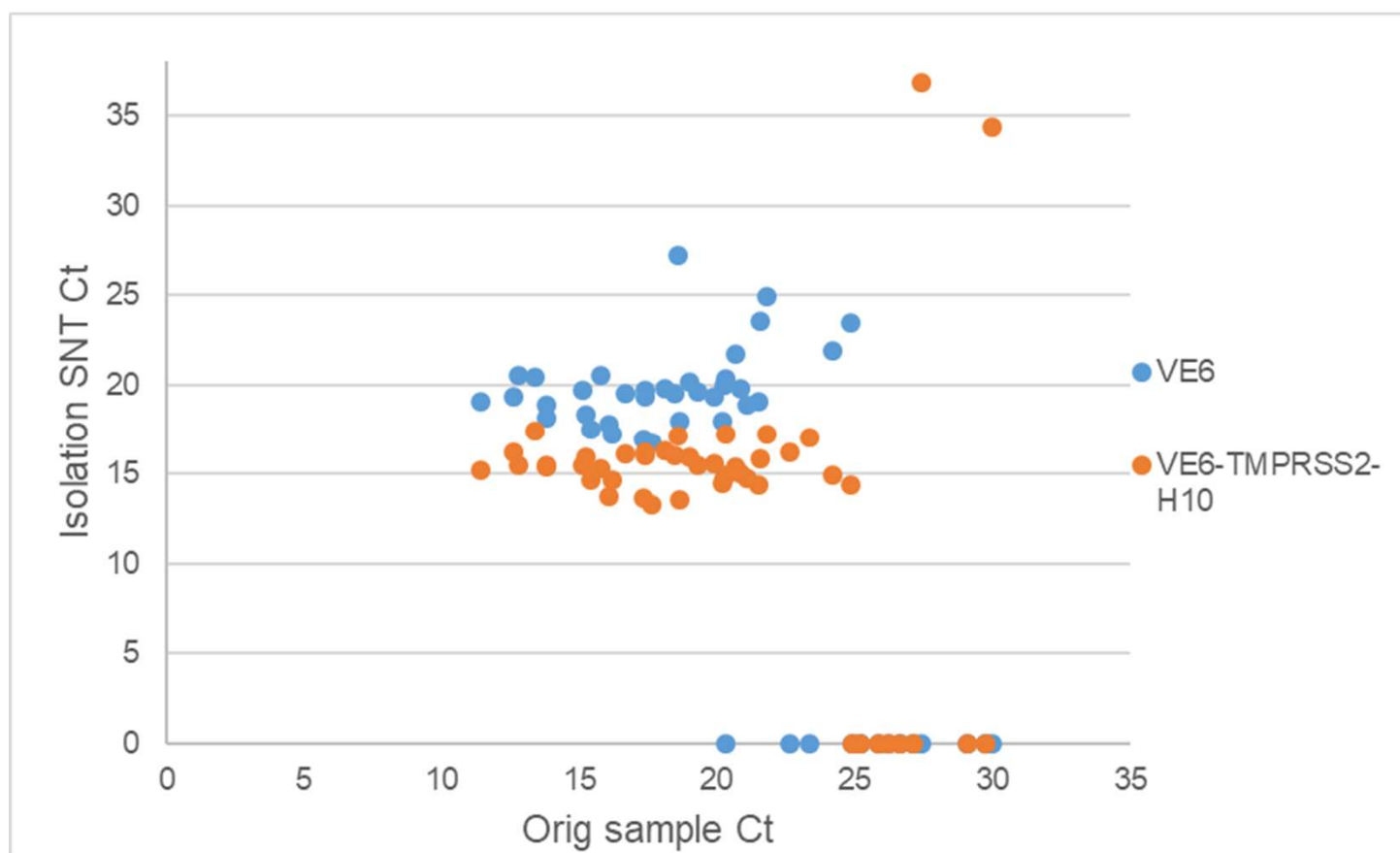

**Figure S9.**

Supplement: FIG S9 [file mbio.00902-21-sf009.pdf]
