# Supplementary material for: A Generic, Scalable, and Rapid Time-Resolved Förster Resonance Energy Transfer-Based Assay for Antigen Detection—SARS-CoV-2 as a Proof of Concept
Source: mBio. 2021 May 18;12(3):e00902-21. doi: 10.1128/mBio.00902-21 (PMC8262888; doi:10.1128/mBio.00902-21)
Supplement: TABLE S1 [file mbio.00902-21-st001.pdf]

HTRF ratio compared to average of mock media 1:1 and 1:100								
	10 µl VE6						10 µl VE6 mock-media	
	1:1	1:10	1:50	1:100	1:1000		1:1	1:100
anti-NP 10 nM Eu + 10 nM AF	18.8	15.9	4.4	2.7	1.2		1.0	1.0
anti-NP 20 nM Eu + 20 nM AF	26.1	12.8	3.7	2.5	1.2		1.0	1.0
anti-NP 40 nM Eu + 40 nM AF	45.0	10.4	2.9	2.1	1.1		1.0	1.0
anti-NP 80 nM Eu + 80 nM AF	39.5	7.0	2.2	1.7	1.1		1.0	1.0
anti-NP 13 nM Eu + 27 nM AF	23.0	16.5	4.5	2.8	1.2		1.0	1.0
anti-NP 27 nM Eu + 13 nM AF	13.0	14.9	4.5	2.8	1.2		1.0	1.0
anti-RBD 10 nM Eu + 10 nM AF	3.6	1.3	1.3	1.1	1.1		1.0	1.0
anti-RBD 20 nM Eu + 20 nM AF	3.3	1.3	1.1	1.0	1.0		1.0	1.0
anti-RBD 40 nM Eu + 40 nM AF	2.7	1.2	1.1	1.0	1.0		1.0	1.0
anti-RBD 80 nM Eu + 80 nM AF	2.3	1.2	1.0	1.0	1.0		1.0	1.0
anti-RBD 13 nM Eu + 27 nM AF	3.1	1.2	1.0	1.0	0.9		1.0	1.0
anti-RBD 27 nM Eu + 13 nM AF	2.3	1.1	1.0	1.1	1.0		1.0	1.0

Table S1.
